# Clinical characteristics and risk factors analysis of *Clostridioides difficile* infection in ulcerative colitis patients during biologic therapy

**DOI:** 10.3389/fcimb.2026.1803104

**Published:** 2026-03-27

**Authors:** Tingting Xu, Ying Xie

**Affiliations:** Department of Gastroenterology, Shengjing Hospital of China Medical University, Shenyang, Liaoning, China

**Keywords:** *Clostridioides difficile* infection, Infliximab, risk factors, ulcerative colitis, Vedolizumab

## Abstract

**Objective:**

To investigate the risk factors for *Clostridioides difficile* infection (CDI) in patients with ulcerative colitis (UC) during biologic therapy.

**Methods:**

Patients diagnosed with UC who received Infliximab (IFX) or Vedolizumab (VDZ) were enrolled. Their clinical characteristics and risk factors for CDI were analyzed.

**Results:**

A total of 110 UC patients treated with IFX (61 cases) or VDZ (49 cases) were included. The overall positive rate of *Clostridioides difficile* (*C. difficile*) was 28.2%. Positive Epstein-Barr virus (EBV)-DNA, a modified Mayo score ≥11 points, and a history of glucocorticoids use within 2 months before admission were independent risk factors for CDI in UC patients receiving IFX/VDZ therapy. The incidence of CDI was the highest within 0–3 months after IFX/VDZ treatment.

**Conclusion:**

Positive EBV-DNA, a modified Mayo score ≥11 points, and a history of glucocorticoids use within 2 months before admission were independent risk factors for CDI in UC patients during IFX/VDZ therapy.

## 1 Introduction

Ulcerative colitis (UC) is an immune-mediated chronic inflammatory bowel disease (IBD) characterized primarily by mucosal leions affecting the rectum and colon. Its therapeutic drugs include aminosalicylate preparations, glucocorticoids, immunosuppressants, biological agents, and small-molecule drugs. Currently, the concept of “step-up” therapy has been downplayed, and biological agents can be used as a first-line treatment regimen for patients with moderate-to-severe active UC (Kucharzik et al., 2021; Inflammatory Bowel Disease Group et al., 2024).The biological agents approved in China for the treatment of UC are Infliximab (IFX) and Vedolizumab (VDZ) (Inflammatory Bowel Disease Group et al., 2024).

*Clostridioides difficile* (*C. difficile*) is a normal component of the intestinal flora. When intestinal microecological imbalance occurs, its dormant spores germinate, proliferate, and release toxins, which can cause *Clostridioides difficile* infection (CDI). This infection not only induces intestinal disorders but also prolongs disease duration or exacerbates the disease. Studies have shown that IBD is an independent risk factor for CDI, with an incidence rate more than five times that of non-IBD patients (Kucharzik et al., 2021). Furthermore, the incidence of CDI in UC patients is higher than that in Crohn’s disease (CD) patients (Khanna, 2021; Spartz et al., 2024). Thus, identifying the risk factors of CDI in UC patients and conducting timely diagnosis and treatment are of great clinical significance.

Therefore, we aimed to explore the clinical characteristics of UC patients and the risk factors for concurrent CDI in the context of biological agent (IFX/VDZ) treatment, so as to improve clinicians’ ability to identify and manage CDI.

## 2 Methods

### 2.1 Study population and design

This study retrospectively enrolled patients diagnosed with UC in the Department of Gastroenterology, Shengjing Hospital of China Medical University, who received IFX/VDZ treatment from January 2019 to May 2025. Time and dosage of biologic agents: IFX: 5 mg/kg was administered intravenously at weeks 0, 2, and 6, followed by maintenance dosing every 8 weeks thereafter. VDZ: 300 mg was administered intravenously at weeks 0, 2, and 6, followed by maintenance dosing every 8 weeks. The study was approved by the Hospital Ethics Committee (Ethics No: 2024PS1598K). All enrolled patients underwent fecal *C. difficile* testing. Patients who were pregnant, lactating, or had a history of active malignant tumors were excluded. General patient data, laboratory tests, disease characteristics [Montreal Classification (Silverberg et al., 2005), Modified Mayo Score (Lobatón et al., 2015), Mayo Endoscopic Score (Schroeder et al., 1987)], concurrent medication use within 2 months before admission, and CDI treatment status were collected. Missing data were handled using multiple imputation. Statistical analysis was performed using SPSS 27.0 software to analyze the clinical characteristics of patients, and logistic regression analysis was used to identify the risk factors for CDI. For variables with a missing data rate of ≥30%, they are directly excluded. For variables with a missing rate of <30%, multiple imputation is used to fill in the missing values. The significance level set at P <0.05.

### 2.2 Diagnostic criteria of *C. difficile*

Fecal samples were collected from patients in this study. Referring to the Chinese Guideline of CDI (Chinese Society of Surgery of Chinese Medical AssociationInfectious Diseases Society for Evidence-based and Translational Medicine of Chinese Research Hospital Association, 2024), nucleic acid amplification test (NAAT) and *C. difficile* toxin B (*tcdB*) gene detection were performed, and *C. difficile* binary toxic (CDT) and *tcd* gene deletion detection were conducted. The result interpretation was as follows: CDI was diagnosed if NAAT (+), *tcdB* gene (+), and the frequency of diarrhea was ≥3 times/day; *C. difficile* colonization was diagnosed if NAAT (+), *tcdB* gene (+), but the frequency of diarrhea was <3 times/day; the presence of a hypervirulent strain was suggested if CDT (+) and *tcd* gene deletion (+), combined with NAAT (+) and *tcdB* gene (+); the absence of CDI was determined if all the above four results were (-).

## 3 Results

### 3.1 Clinical characteristics of the study population

A total of 110 patients with UC were enrolled in this study. The positive rate of *C. difficile* was 28.2%, including a *C. difficile* infection rate of 23.6% and a colonization rate of 4.5%. Among all infected patients, only one case was infected with a hypervirulent strain. According to the type of biological agent used, 61 patients received IFX and 49 received VDZ.

The clinical characteristics of the patients were as follows: 71 males and 39 females, with a male-to-female ratio of 1.82:1; the age ranged from 14 to 82 years, with a mean age of 48.43 ± 16.39 years; the mean length of hospital stay was 10.98 ± 7.67 days; the mean BMI was 20.23 ± 3.28 kg/m²; the mean disease duration was 61.85 ± 62.07 months. There were 28 smokers and 28 drinkers; 16 patients had hypertension, 7 had diabetes, and 26 (23.6%) had a previous history of CDI. Seventy-six patients (69.1%) were treatment-naïve, and 34 had received prior biologic therapy. In terms of clinical manifestations, 81 patients (73.6%) had abdominal pain, 82 (74.5%) had bloody stools, while only 13 patients (11.8%) presented with fever. In total, 14 patients tested positive for EBV-DNA. Regarding disease-related data: 54.5% (60 patients) had severe disease activity according to the modified Mayo score; 85.5% of UC patients had a Mayo endoscopic subscore of 3 (severe active phase); extensive colonic involvement was observed in 80.6% (89 patients) of cases. Within 2 months before admission, the most frequent combination medication was corticosteroids, used in 32 patients (29.1%). Detailed data are shown in **Table 1**.

**Table 1:** Comparison of clinical characteristics between patients in CDI group and non-CDI group.

Items	Total(n=110)	CDI group (n=26)	Non-CDI group (n=84)	P value
General characteristics				
Sex	Male 71/110(64.5%) Female 39/110(35.5%)	17/26(65.4%) 9/26(34.6%)	54/84(64.3%) 30/84(35.7%)	0.918
Age(years)	48.43 ± 16.39	47.12 ± 15.04	48.83 ± 16.85	0.643
Length of hospital stay (days)	10.98 ± 7.67	13.62 ± 8.79	10.17 ± 7.14	0.044
BMI(kg/m^2^)	20.23 ± 3.28	19.88 ± 3.13	20.34 ± 3.33	0.535
Smoking history	28/110(25.5%)	8/26(30.8%)	20/84(23.8%)	0.477
Alcohol consumption history	28/110(25.5%)	10/26(38.5%)	18/84(21.4%)	0.081
Total disease duration (months)	61.85 ± 62.07	56.08 ± 54.49	63.63 ± 64.44	0.590
Hypertension	16(14.5%)	3 (11.5%)	13(15.5%)	0.858
Diabetes mellitus	7/110(6.4%)	0	7/84(8.3%)	0.288
Previous CDI history	26/110(23.6%)	7/26(26.9%)	18/84(21.4%)	0.559
Initial treatment	76/110(69.1%)	16/26(61.5%)	60/84(71.4%)	0.340
Abdominal pain	81/110(73.6%)	19/26(73.1%)	62/84(73.8%)	0.941
Fever	13/110(11.8%)	7/26(26.9%)	6/84(7.1%)	0.017
Hematochezia	82/110(74.5%)	19/26(73.1%)	63/84(75%)	0.844
Laboratory examinations				
C-reactive protein	29.40 ± 40.51	36.54 ± 48.00	27.19 ± 37.95	0.306
White blood cell count	7.97 ± 3.28	7.99 ± 2.11	7.96 ± 3.58	0.968
Neutrophil count	5.04 ± 2.93	5.24 ± 1.86	4.97 ± 3.19	0.682
Neutrophil-to-lymphocyte ratio	3.18 ± 3.08	3.67 ± 2.56	3.03 ± 3.23	0.356
Hemoglobin	112.21 ± 25.71	115.62 ± 27.38	111.15 ± 25.25	0.442
Platelet count	316.36 ± 133.70	303.69 ± 150.95	320.29 ± 128.64	0.583
Albumin	34.80 ± 5.68	34.92 ± 5.65	34.76 ± 5.72	0.900
Creatinine	66.73 ± 15.00	69.00 ± 13.23	66.04 ± 15.52	0.381
Alanine aminotransferase	10(7, 15)	11(8, 19.75)	9.5(7, 15)	0.116
Aspartate aminotransferase	13(10,19)	13(10, 18.75)	13.5(10.75, 21.25)	0.297
Total cholesterol	3.94 ± 0.94	3.92 ± 1.01	3.95 ± 0.93	0.908
Triglyceride	1.23 ± 0.72	1.31 ± 0.90	1.20 ± 0.67	0.489
D-dimer	293.87 ± 369.30	303.15 ± 239.41	291 ± 402.25	0.884
Dominant intestinal flora	G+bacilli 28/110(25.5%) G-bacilli 71/110(64.5%) G+cocci 11/110(10.0%)	5/26(19.2%) 19/26(73.1%) 2/26(7.7%)	23/84(27.4%) 52/84(61.9%) 9/84(10.7%)	0.572
Total fecal bacteria count	<10 15/110(13.6%) 11-100 23/110(20.9%) 101-500 37/110(33.6%) 501-5000 33/110(30.0%) >5000 2/110(1.8%)	1/26(3.8%) 5/26(19.2%) 10/26(38.5%) 9/26(34.6%) 1/26(3.8%)	14/84(16.7%) 18/84(21.4%) 27/84(32.1%) 24/84(28.6%) 1/84(1.2%)	0.377
EBV-DNA	Positive 14/110(12.7%) Negative 96/110(87.3%)	7/26(26.9%) 19/26(73.1%)	7/84(8.3%) 77/84(91.7%)	0.032
Disease-related characteristics				
Modified Mayo score	Clinical remission 3/110(2.7%) Mild activity 10/110(9.1%) Moderate activity 37(33.6%) Severe activity 60(54.5%)	0 0 6/26(23.1%) 20/26(76.9%)	3/84(3.6%) 10/84(11.9%) 31/84(36.9%) 40/84(47.6%)	0.047
Mayo endoscopic score	Score 0 3/110(2.7%) Score 1 2/110(1.8%) Score 2 11/110(10%) Score 3 94/110(85.5%)	0 0 3/26(11.5%) 23/26(88.5%)	3/84(3.6%) 2/84(2.4%) 8/84(9.5%) 71/84(84.5%)	1.000
Montreal classification	E1 2/110(1.8%) E2 19/110(17.3%) E3 89/110(80.9%)	0 4/26(15.4%) 22/26(84.6%)	2/84(2.4%) 15/84(17.9%) 67/84(79.7%)	1.000
Concomitant medications within 2 months before admission				
Antibiotics	9/110(8.2%)	3/26(11.5%)	6/84(7.1%)	0.760
Immunosuppressants	8/110(7.3%)	2/26(7.7%)	6/84(7.1%)	1.000
glucocorticoids	32/110(29.1%)	13/26(50%)	19/84(22.6%)	0.007
Wuwei Kushen Enteric-coated Capsules	11/110(10%)	5/26(19.2%)	6/84(7.1%)	0.155
Aminosalicylates	23/110(20.9%)	7/26(26.9%)	16/84(19%)	0.388
Proton pump inhibitors	24/110(21.8%)	8/26(30.8%)	16/84(19.0%)	0.206

In the between-group comparison (see **Table 1** for details): the length of hospital stay was significantly longer in the CDI group than in the non-CDI group. The proportion of patients with fever (26.9%) was significantly higher in the CDI group than in the non-CDI group (7.1%). In addition, the percentage of patients with positive EBV-DNA was also significantly higher in the CDI group. In terms of disease characteristics, 76.9% of patients in the CDI group had severe disease activity based on the modified Mayo score. With regard to medication history, the proportion of patients with corticosteroids use within 2 months before admission was higher in the CDI group than in the non-CDI group, with P < 0.05.

### 3.2 Analysis of risk factors for CDI in UC patients treated with IFX/VDZ

Results of univariable analysis showed that positive EBV-DNA, a modified Mayo score ≥11 points, and a history of glucocorticoids use within 2 months before admission might increase the risk of CDI. For UC patients receiving IFX or VDZ treatment who developed concurrent CDI, the condition might lead to prolonged hospital stays and fever. Considering that prolonged hospital stay and fever were merely outcomes of CDI, only positive EBV-DNA, a modified Mayo score ≥11 points, and a history of glucocorticoids use within 2 months before admission were included in the multivariable analysis. Multivariable analysis revealed that positive EBV-DNA, a modified Mayo score ≥11 points, and a history of glucocorticoids use within 2 months before admission were independent risk factors for CDI in UC patients treated with IFX/VDZ. For details, see **Table 2**.

**Table 2:** Analysis of risk factors for CDI in UC patients treated with IFX/VDZ.

Variables	Univariable Analysis		Multivariable Analysis	
	Odds Ratio(95%CI)	*P* Value	Odds Ratio(95%CI)	*P* Value
Length of hospital stay	2.88(1.17-7.10)	0.022		
Symptom-fever	4.79(1.44-15.90)	0.011		
Positive EBV-DNA	4.05(1.27-12.95)	0.018	4.416(1.26-15.80)	0.020
A modified Mayo score ≥11 points	3.67(1.34-10.05)	0.012	3.56(1.22-10.39)	0.020
History of glucocorticoids within 2 months before admission	3.42(1.36-8.61)	0.009	3.10(1.16-8.28)	0.024

The length of hospital stay was set with <10 days as the reference; Fever was set with no fever as the reference; Positive EBV-DNA was set with negative EBV-DNA as the reference; A modified Mayo score ≥11 points was set with <11 points as the reference;History of glucocorticoids use within 2 months before admission was set with no glucocorticoids use as the reference.

### 3.3 Comparison of the epidemiology of CDI and the onset time following the administration of biologic agents between the VDZ group and the IFX group

There were 61 cases in the IFX group and 49 cases in the VDZ group. In the IFX group, the *C. difficile* positivity rate was 23% and the infection rate was 18%; in the VDZ group, the *C. difficile* positivity rate was 34.7% and the infection rate was 30.6%. No statistically significant differences were observed in either the positivity rate or the infection rate between the two groups. Analysis of the onset time of CDI in patients of the two groups showed that among UC patients, the incidence of CDI was the highest within 0–3 months after the initiation of VDZ/IFX treatment; however, this difference was not statistically significant. For details, see **Table 3**.

**Table 3:** Comparison of the epidemiology of CDI and the onset time following the administration of biologic agents between the VDZ group and the IFX group.

Items		VDZ Group	IFX Group	*χ²*	*P* Value
*C. difficile* Positivity Rate	Positive 31/110/(28.2%)	17/49(34.7%)	14 /61(23%)	1.851	0.174
*C. difficile* Infection Rate	Infection 26/110(23.6%)	15/49(30.6%)	11/61(18%)	2.382	0.123
Onset Time of CDI n(%)	≤3 months 19/26(73.1%) 4–12 months 3/26(11.5%) >12 months 4/26(15.4%)	9/15(60%) 3/15(20%) 3/15(20%)	10/11(90.9%) 0 1/11(9.1%)	3.054	0.296

### 3.4 Treatment details of CDI

A total of 31 patients with positive *C. difficile* test results were included in this study, consisting of 26 patients with confirmed infection (CDI) and 5 patients with colonization. Among the CDI patients, 23 received oral vancomycin treatment; 1 patient was switched to oral metronidazole due to vancomycin intolerance; 1 patient with recurrent CDI underwent fecal microbiota transplantation (FMT) after evaluation; another patient was discharged against medical advice without treatment due to personal reasons. Among the colonized patients, 2 received no treatment; 3 received oral vancomycin.

When stratified by infection type: 21 cases (80.8%) were primary CDI; 19 received oral vancomycin, 1 was switched to oral metronidazole due to vancomycin intolerance, and 1 was discharged against medical advice without treatment; 5 cases (19.2%) were recurrent CDI; 1 underwent FMT, and the remaining 4 all received oral vancomycin treatment.

## 4 Discussion

A real-world study showed the incidence of CDI was 20.6% in the IFX group and 4.8% in the VDZ group among UC patients (Zhao, 2024); another study indicated the incidence of CDI after VDZ treatment was only 6.2% (Li et al., 2025). Additionally, foreign scholars demonstrated the *C. difficile* positivity rate in IBD patients who had used IFX within 1 year was 34.8% (Anderson et al., 2017), and existing studies have confirmed that UC patients treated with VDZ had a lower CDI risk than those treated with anti-tumor necrosis factor agents (Chen et al., 2024). In this study, the *C. difficile* positivity rate in IFX-treated UC patients was consistent with previous studies; however, that in VDZ-treated patients was significantly higher, conflicting with previous research conclusions. The putative reasons include this study’s small sample size, the inclusion of patients with a previous history of CDI (increasing the risk of reinfection), and regional differences in baseline characteristics and diagnosis or treatment practices.

The manifestations of IBD complicated with CDI are highly similar to those of active IBD, making clinical differentiation often difficult. In this study, when comparing clinical manifestations and laboratory indicators, patients in the CDI group had a longer length of hospital stay and a higher proportion of fever. This conclusion was consistent with previous clinical experience. Furthermore, this study confirmed that positive EBV-DNA was a risk factor for CDI in UC patients receiving biologic agents. When IBD patients present with aggravated diarrhea accompanied by systemic symptoms such as fever and pharyngitis, EBV infection warrants attention; a high or dynamically increasing EBV-DNA load can serve as an indicator. To date, no studies have reported the association between the two. EBV infection may indirectly increase the risk of CDI by inducing immune dysfunction, exacerbating the underlying disease, or disrupting intestinal microecological balance. However, this study did not further investigate the impact of EBV infection on disease severity and immune status. The potential association and molecular mechanisms between them still need to be further clarified with an expanded sample size and combined basic experiments. Although studies have shown that IBD patients complicated with CDI exhibited characteristic differences in the species composition of intestinal flora (Yu et al., 2023), this study only preliminarily determined the presence of intestinal dysbiosis by detecting the Gram staining characteristics of patients’ intestinal flora, and no statistically significant difference was observed between the two groups. To analyze the structure and changes of intestinal flora more accurately, further studies using molecular biological techniques such as rRNA sequencing are required.

This study confirmed that a modified Mayo score ≥11 points was an independent risk factor for CDI in UC patients receiving IFX/VDZ treatment. This was consistent with the conclusions of previous studies that patients with active IBD have a higher risk of CDI (Consensus Expert Group of Journal of Modern Digestion & Intervention, 2019; Drozdinsky et al., 2023). The possible mechanism is that the intestinal barrier is impaired during the active phase, which facilitates the colonization of *C. difficile*. Although meta-analyses have indicated that colonic involvement is a risk factor for CDI in IBD patients (Balram et al., 2019; Zhang, 2020), there was no significant difference in the proportion of colonic involvement between the two groups in this study. This is presumably related to the relatively severe overall condition of the patients included in this study. In the future, the sample size needs to be expanded to further reduce selection bias and explore the association between the two more accurately.

This study indicated that the use of glucocorticoids within 2 months before admission increased the risk of CDI in patients with UC, which was consistent with the conclusions of previous studies (Jakubowska et al., 2024). However, although it is widely accepted that the combination of biologics with antibiotics and immunosuppressants significantly increases the risk of CDI (Zhang et al., 2017; Gholam-Mostafaei et al., 2020), and that PPI use is also associated with an elevated risk of CDI (Eeuwijk et al., 2024; Finke et al., 2025), but this study did not observe any association between these factors and CDI. This may be due to the limited sample size, recall bias, and documentation bias. Future large-sample, multicenter prospective studies with standardized, real-time, and comprehensive medical records are warranted to collect accurate information on patients.

There are few specific studies on the onset time of CDI in IBD patients after initiating biologic therapy at home and abroad, but relevant studies can provide references: the risk of infection was the highest in the early stage of anti-TNF agent use (Ma et al., 2023), and the risk of opportunistic infection in rheumatoid arthritis patients receiving biologic therapy in the short term (< 6 months) was higher than that in the long-term stage (Hashimoto et al., 2017).These findings lay an important foundation for this study. This study showed that among UC patients receiving IFX/VDZ treatment, the incidence of CDI was the highest within 0–3 months after the start of treatment. This was basically consistent with the conclusions of previous studies. This result also sounds an alarm for clinical practice: in the early stage of biologic therapy, it is necessary to strengthen the monitoring of CDI to detect and take intervention measures in a timely manner.

The treatment of CDI in this study mainly focused on vancomycin, metronidazole, and fecal microbiota transplantation. The Chinese guideline (Chinese Society of Surgery of Chinese Medical AssociationInfectious Diseases Society for Evidence-based and Translational Medicine of Chinese Research Hospital Association, 2024) acknowledges the efficacy of vancomycin and metronidazole; The 2021 IDSA guideline (Johnson et al., 2021) recommends fidaxomicin and vancomycin for the treatment of CDI, with metronidazole as an alternative option. FMT is recommended for patients with ≥2 recurrences or those who have failed antibiotic therapy. Domestic and international guidelines are generally consistent. Although the Chinese guideline also recognizes the efficacy of fidaxomicin, its limited availability in China restricts its use to patients with recurrence or at risk of recurrence only when the drug is accessible. Therefore, fidaxomicin was not used in our hospital.

This study was a single-center retrospective study with certain limitations. The late implementation of routine testing for *C. difficile* in our hospital resulted in a limited sample size, which may affect the stability and representativeness of the findings. During the follow-up period of biologic therapy, infection-related screenings such as *C. difficile* and EBV-DNA testing were not performed at every hospital admission, which may lead to missing data. In addition, this study only included patients who underwent CDI testing, while those who did not receive CDI testing were excluded, resulting in selection bias that may impact the study results. Future multicenter, large-sample prospective studies are warranted to further validate and refine the conclusions of this study, and improve the reliability and generalizability of the results.

## 5 Conclusions

Positive EBV-DNA, a modified Mayo score ≥11 points, and a history of glucocorticoids use within 2 months before admission were independent risk factors for CDI in UC patients during IFX/VDZ therapy.

## Data Availability

The raw data supporting the conclusions of this article will be made available by the authors, without undue reservation.

## References

[B1] Anderson A. Click B. Ramos-Rivers C. Cheng D. Babichenko D. Koutroubakis I. . (2017). Lasting impact of *Clostridium difficile* infection in inflammatory lasting impact of *Clostridium difficile* infection in inflammatory bowel disease: A propensity score matched analysis. Inflamm Bowel Dis. 23, 2180–2188. doi: 10.1097/MIB.0000000000001251. PMID: 29084081 PMC5685936

[B2] Balram B. Battat R. Al-Khoury A. D'Aoust J. Afif W. Bitton A. . (2019). Risk factors associated with *Clostridium difficile* infection in inflammatory bowel disease: A systematic review and meta-analysis. J. Crohn's Colitis 13, 27–38. doi: 10.1093/ecco-jcc/jjy143. PMID: 30247650

[B3] Chen W. Liu Y. Zhang Y. Zhang H. Chen C. Zhu S. . (2024). Risk of *Clostridioides difficile* infection in inflammatory bowel disease patients undergoing vedolizumab treatment: A systematic review and meta-analysis. BMC Gastroenterol. 24, 377. doi: 10.1186/s12876-024-03460-z. PMID: 39448963 PMC11515399

[B4] Chinese Society of Surgery of Chinese Medical AssociationInfectious Diseases Society for Evidence-based and Translational Medicine of Chinese Research Hospital Association (2024). Guidelines for diagnosis, treatment and prevention of *Clostridium difficile* infection in China (2024). Zhonghua Wai Ke Za Zhi 62, 893–908. doi: 10.3760/cma.j.cn112139-20240528-00259. PMID: 39183013

[B5] Consensus Expert Group of Journal of Modern Digestion & Intervention (2019). Expert consensus on the management of adult inflammatory bowel disease complicated with *Clostridioides difficile* infection in China. Mod Digest Intervent 24, 448–452.

[B6] Drozdinsky G. Atamna A. Banai H. Ben-Zvi H. Bishara J. Eliakim-Raz N. (2023). Clinical outcomes for *Clostridioides difficile* associated diarrhea in inflammatory bowel disease patients versus non-IBD population: A retrospective cohort study. Medicine 102, e32812. doi: 10.1097/MD.0000000000032812. PMID: 36820599 PMC9907955

[B7] Eeuwijk J. Ferreira G. Yarzabal J. P. Robert-Du Ry Van Beest Holle M. (2024). A systematic literature review on risk factors for and timing of *Clostridioides difficile* infection in the United States. Infect. Dis. Ther. 13, 273–298. doi: 10.1007/s40121-024-00919-0. PMID: 38349594 PMC10904710

[B8] Finke M. Boven A. Vlieghe E. Engstrand L. Orsini N. Brusselaers N. (2025). Proton pump inhibitors and the risk of *Clostridioides difficile* infection: A systematic review and dose-response meta-analysis. J. Infect 90, 106488. doi: 10.1016/j.jinf.2025.106488. PMID: 40239816

[B9] Gholam-Mostafaei F. S. Yadegar A. Aghdaei H. A. Azimirad M. Daryani N. E. Zali M. R. (2020). Anti-TNF containing regimens may be associated with increased risk of *Clostridioides difficile* infection in patients with underlying inflammatory bowel disease. Curr. Res. Trans. Med. 68, 125–130. doi: 10.1016/j.retram.2020.03.002. PMID: 32414632

[B10] Hashimoto A. Suto S. Horie K. Fukuda H. Nogi S. Iwata K. . (2017). Incidence and risk factors for infections requiring hospitalization, including Pneumocystis pneumonia, in Japanese patients with rheumatoid arthritis. Int. J. Rheumatol. 2017, 6730812. doi: 10.1155/2017/6730812. PMID: 29181029 PMC5664349

[B11] Inflammatory Bowel Disease Group Chinese Society of Gastroenterology Chinese Medical AssociationInflammatory Bowel Disease Quality Control Center of China (2024). 2023 Chinese national clinical practice guideline on diagnosis and management of ulcerative colitis. Chin. Med. J. 137, 1642–1646. doi: 10.1097/CM9.0000000000003221. PMID: 38955435 PMC11268805

[B12] Jakubowska A. Szydlarska D. Rydzewska G. (2024). Analysis of risk factors of *Clostridioides difficile* infection in patients with inflammatory bowel disease. Przegl Gastroenterol 19, 277–283. doi: 10.5114/pg.2024.143145. PMID: 39802969 PMC11718504

[B13] Johnson S. Lavergne V. Skinner A. M. Gonzales-Luna A. J. Garey K. W. Kelly C. P. . (2021). Clinical practice guideline by the Infectious Diseases Society of America (IDSA) and Society for Healthcare Epidemiology of America (SHEA): 2021 focused update guidelines on management of *Clostridioides difficile* infection in adults. Clin. Infect. Dis. 73, e1029–e1044. doi: 10.1093/cid/ciab549. PMID: 34164674

[B14] Khanna S. (2021). Management of *Clostridioides difficile* infection in patients with inflammatory bowel disease. Intestinal Res. 19, 265–274. doi: 10.5217/ir.2020.00045. PMID: 32806873 PMC8322030

[B15] Kucharzik T. Ellul P. Greuter T. Rahier J. F. Verstockt B. Abreu C. . (2021). ECCO guidelines on the prevention, diagnosis, and management of infections in inflammatory bowel disease. J. Crohn's Colitis 15, 879–913. doi: 10.1093/ecco-jcc/jjab052. PMID: 33730753

[B16] Li Q. Song X. Su P. Lv X. Liu X. Chen X. . (2025). Risk factors and clinical characteristics of *Clostridium difficile* colonization and infection in patients with inflammatory bowel disease exposed to Vedolizumab: A multicenter retrospective study. Ther. Adv. Gastroenterol. 18, 17562848251321707. doi: 10.1177/17562848251321707. PMID: 39975482 PMC11837063

[B17] Lobatón T. Bessissow T. De Hertogh G. Lemmens B. Maedler C. Van Assche G. . (2015). The Modified Mayo Endoscopic Score (MMES): A new index for the assessment of extension and severity of endoscopic activity in ulcerative colitis patients. J. Crohn's Colitis 9, 846–852. doi: 10.1093/ecco-jcc/jjv111. PMID: 26116558

[B18] Ma X. J. Liu X. Ge Y. (2023). Pay attention to the infectious complications in the clinical application of biological agents. Zhonghua Yi Xue Za Zhi 103, 2546–2551. doi: 10.3760/cma.j.cn112137-20230608-00962. PMID: 37650201

[B19] Schroeder K. W. Tremaine W. J. Ilstrup D. M. (1987). Coated oral 5-aminosalicylic acid therapy for mildly to moderately active ulcerative colitis. A randomized study. N. Engl. J. Med. 317, 1625–1629. doi: 10.1056/NEJM198712243172603. PMID: 3317057

[B20] Silverberg M. S. Satsangi J. Ahmad T. Arnott I. D. Bernstein C. N. Brant S. R. . (2005). Toward an integrated clinical, molecular and serological classification of inflammatory bowel disease: Report of a Working Party of the 2005 Montreal World Congress of Gastroenterology. Can. J. Gastroenterol. 19, 5A–36A. doi: 10.1155/2005/269076. PMID: 16151544

[B21] Spartz E. J. DeDecker L. C. Fansiwala K. M. Noorian S. Roney A. R. Hakimian S. . (2024). Recent trends and risk factors associated with *Clostridioides difficile* infections in hospitalized patients with inflammatory bowel disease. Aliment Pharmacol. Ther. 59, 89–99. doi: 10.1111/apt.17777. PMID: 37873878

[B22] Yu S. Li Y. Xu H. Tian B. W. Shi Y. J. Qian J. M. (2023). Analysis of gut microbiota in inflammatory bowel disease patients with *Clostridium difficile* infection. Chin. J. Inflamm. Bowel Dis. 07, 48–54. doi: 10.3760/cma.j.cn101480-20220324-00049. PMID: 40668938

[B23] Zhang Y. J. (2020). “ Risk factors for inflammatory bowel disease combined with *Clostridium difficile* infection: A meta-analysis,” in Department of Gastroenterology (China: Shanxi Medical University).

[B24] Zhang T. Lin Q. Y. Fei J. X. Zhang Y. Lin M. Y. Jiang S. H. . (2017). Corrigendum: *Clostridium difficile* infection worsen outcome of hospitalized patients with inflammatory bowel disease. Sci. Rep. 7, 43975. doi: 10.1038/srep43975. PMID: 28332614 PMC5362761

[B25] Zhao Z. C. (2024). A real-world study of secondary *Clostridium difficile* infection in patients with moderate-to severe ulcerative colitis treated with infliximab and vedolizumab (China: Hebei Medical University).

